# 

**Published:** 1860-01

**Authors:** 


*New Publications Received.*

The Diagnosis, Pathology, and Treatment of the Diseases of the Chest. By W. W. GERHARD, M. D., one of the Physicians of the Pennsylvania Hospital ; Fellow of the College of Physicians of Philadelphia ; Member of the American Philosophical Society. Fourth edition, revised and enlarged. Philadelphia : J. B. Lippincott & Co.

The Obstetric Catechism ; containing two thousand three hundred and forty-seven questions and answers on Obstetrics proper. By JOSEPH WARRINGTON, M. D. One hundred and fifty illustrations. Philadelphia: J. B. Lippincott & Co.

On the Formation of Artificial Anus. By C. W. MEIER, M. D., Surgeon to Bellevue Hospital.

On the Effect of Pressure on Ulcerated Vertebræ, and in Morbus Coxarius, and the relief afforded by Mechanical Remedies, with Cases. By H. G. DAVIS, M. D., 67 Union Place, New York.

Ancient Marriages of Consanguinity. By ISAAC CASSELBERRY, M. D. From the Nashville Journal of Medicine and Surgery.

---

☞ Prof. AUSTIN FLINT has now left New York, to perform the duties of his Chair in the New Orleans School of Medicine. He will return early next spring; and until then, correspondents will please address him at New Orleans.

Our office and residence is still at 111 East 21st st., Gramercy Park House, where letters to us should be addressed.

